# Antitumor Activity of *Citrus maxima* (Burm.) Merr. Leaves in Ehrlich's Ascites Carcinoma Cell-Treated Mice

**DOI:** 10.5402/2011/138737

**Published:** 2011-04-19

**Authors:** Sriparna KunduSen, Malaya Gupta, Upal K. Mazumder, Pallab K. Haldar, Prerona Saha, Asis Bala

**Affiliations:** Department of Pharmaceutical Technology, Jadavpur University, Kolkata, West Bengal 700032, India

## Abstract

*Context*. The plant *Citrus maxima* Merr. (Rutaceae), commonly known as shaddock or pomelo is indigenous to tropical parts of Asia. The objective of present study is to evaluate the methanol extract of *Citrus maxima* leaves for its antitumor activity against Ehrlich's Ascites Carcinoma cell in Swiss albino mice. *Experimental design*. The antitumor activity of methanol extract of *Citrus maxima* leaves (MECM) was evaluated against Ehrlich Ascites Carcinoma (EAC) cell line in Swiss albino mice. 2 × 10^6^ cells were inoculated in different groups of animals. MECM (200 and 400 mg/kg BW i.p.) was administered for nine consecutive days. On day 10th half the animals of different groups were sacrificed for determination of tumor and haematological parameters and the rest half were kept with sufficient food and water ad libitum for determination of increase in life span. *Result and Discussions*. Oral administration of the extract at the doses of 200 and 400 mg/kg significantly decreased tumor parameters such as tumor volume, viable tumor cell count and increased body weight, hematological parameters and life span in respect of the EAC control mice. *Conclusion*. Experimental design exhibits significant antitumor activity of the extract (MECM) in a dose dependant manner.

## 1. Introduction

Cancer, otherwise termed as Malignant Neoplasm, is a class of diseases in which a group of cells show abnormal proliferation of cells, invasion and sometimes metastasis. These three are the characters that differentiate malignant neoplasms from benign tumors that they do not divide abnormally beyond normal limits, do not invade or metastasize [1]. The aim of current research has been the identification of natural and synthetic compounds that can be used in the prevention and/or treatment of cancer. Plants have been regarded as a potential source of cancer chemoprevention drug discovery and development [2].

*Citrus maxima *and *C. grandis* (family: Rutaceae) are both currently used for the shaddock or pomelo. Although *C. grandis* (L.) Osbeck is more frequently used *C. maxima* (J. Burman) Merrill is correct under the International Code of Botanical Nomenclature [3].

The pulp is stated to possess the following properties as reported in ancient and medieval literature: appetizer, antitoxic, cardiac stimulant, and stomach tonic [4].

The major flavanones of pomelo are neohesperidin and naringin, which are high in the seed in case of unripe citrus fruits [5] and its extract showed antioxidant activity through free radical-scavenging in vitro and to reduce reactive oxygen species in H_2_O_2_-treated HepG2 cells [6]. DPPH free radical scavenging activity and ferric-reducing antioxidant power values determined for the essential oil were 26.1 ± 1.2% and 2.3 ± 0.3 mM, respectively, which were significantly higher than those of various fruit pulp extracts [7]. Hesperidin and naringin were present in fruit juice. Caffeic, p-coumaric, ferulic, and vanillic acid are also present in the fruit juice [8]. A C-C linked bisacridone alkaloid, buntanbismine, was isolated from the stem bark of *C. grandis *[9]. *C. maxima* essential oil is composed of *α*-Pinene, Sabenine, *β*-Pinine, Methyl heptenone, *β*-myrcene, Hexanal, Sabenine, DL-Limonene, t-Ocimene, Linalool, 1-Hexene, 4-methyl; 1-Hexene,3,3-dimethyl; Geranyl formate, Z-Citral, Geranyl formate, E-Citral, Geranyl acetate, *β*-Farnesene, and at 750 ppm and 1000 ppm it inhibited mycelia growth of *A. flavus* [10]. The antiproliferative effect of an immature *C. grandis* fruit extract was investigated using U937 human leukaemia cells. The induction of apoptosis was confirmed by caspase-3 activity assays and by immunoblotting using antibodies against Bcl-2, Bax, poly(ADPribose) polymerase, caspase-9, and caspase-3 [11]. 

## 2. Materials and Methods

### 2.1. Plant Material

The leaves of *C. maxima* were dried under shade and powdered by mechanical grinder. About 500 g of the plant material was successively extracted with petroleum ether and methanol in a Soxhlet apparatus. The methanol was then evaporated under reduced pressure to get the crude extract (MECM, yield 18.1%).

### 2.2. Phytochemical Screening

The plant extracts were subjected to screening for various phytochemicals employing standard protocol for determining the presence of steroids, alkaloids, tannins, flavonoids, glycosides, and so forth [12]. 

### 2.3. Chemicals

Sodium chloride, trypan blue, methyl violet, methylene blue, 5-fluorouracil (Merck ltd., Mumbai, India). All other reagents used were of the highest analytical grade. 

### 2.4. Acute Toxicity

 The acute toxicity of the extract was determined according to the OECD guideline no. 420. Male albino mice weighing 27–30 g were used for this study. MECM was given to four groups (*n* = 5) of animals at 5, 50, 300, and 2000 mg/kg BW p.o. The treated animals were under observation for 14 days, for mortality and general behaviour. No death was observed till the end of the study. The test samples were found to be safe up to the dose of 2000 mg/kg. 

### 2.5. Animals

Male Swiss albino mice weighing 20–22 g were used for present investigation. They were obtained from B. N. Ghosh and Co., Kolkata. They were acclimatized to the laboratory conditions prior to the study for seven days. The animals were kept at 25 ± 2°C and a relative humidity of 40–45% with alternative day and night cycles of 12 hours each. The animals had free access to pellet food (Hindustan Lever, Mumbai, India) and water *ad libitum*. 

### 2.6. Transplantation of Tumor

EAC cells were obtained from Chittaranjan National Cancer Institute (CNCI), Kolkata, India. The EAC cells were maintained in *in vivo* in Swiss albino mice by peritoneal transplantation of 2 × 10^6^ cells per mouse every 10 days. Ascitic fluid was drawn from tumor-bearing mice at the log phase (days 7-8 of tumor bearing) of the tumor cells. Each animal received 0.1 mL of tumor cell suspension containing 2 × 10^6^ tumor cells i.p. 

### 2.7. Treatment Schedule

60 Swiss albino mice were divided into five groups (*n* = 12). All groups except Group I received EAC cells (2 × 10^6^ cells/mouse i.p.) and this was taken as the 0th day. Group I served as saline control (5 mL/kg 0.9% NaCl w/v p.o). Group II served as EAC control. Twenty-four hours after EAC transplantation, Groups III and IV received MECM (200 and 400 mg/kg BW i.p.), and Group V received reference drug 5-fluorouracil (5-FU, 20 mg/kg BW i.p.) daily for nine consecutive days [13]. Twenty-four hours of last dose and 18 h of fasting, six animals of each group were sacrificed by cervical dislocation for measurement of tumor and biochemical parameters, and the rest were kept with food and water *ad libitum* to check the percentage increase in life span. Blood was collected by cardiac puncture for estimation of the haematological parameters. The antitumor activity of the MECM was measured in EAC animals with respect to the following parameters. 

### 2.8. Tumor Volume

The ascitic fluid was collected from the peritoneal cavity, and volume was measured by taking it in a graduated centrifuge tube. 

### 2.9. Percentage Increase in Life Span

The effect of MECM on percentage increases in life span was calculated on the basis of mortality of the experimental mice [13].

(1) $$\begin{array}{c} \text{ILS}\left(\text{\%}\right)=\left(\frac{\text{mean}\;\text{survival}\;\text{time}\;\text{of}\;\text{treated}\;\text{group}}{\text{mean}\;\text{survival}\;\text{time}\;\text{of}\;\text{control}\;\text{group}}-1\right)\times 100,\\ \text{Mean}\;\text{survival}\;{\text{time}}^{*}=\frac{\text{first}\;\text{death}+\text{last}\;\text{death}}{2}. \end{array}$$

*Time is denoted by days. 

### 2.10. Tumor Cell Count

The ascitic fluid was taken in a WBC pipette and diluted to 100 times. Then a drop of the diluted cell suspension was placed on the Neubauer's counting chamber, and the numbers of cells in the 64 small squares were counted. 

### 2.11. Viable/Nonviable Tumor Cell Count

The viability and nonviability of the cell were checked by trypan blue assay. The cells were stained with trypan blue (0.4% in normal saline) dye. The cells that did not take up the dye were viable, and those that took the dye were nonviable. These viable and nonviable cells were counted.

(2) $$\text{Cell}\;\text{counter}=\frac{\text{Number}\;\text{of}\;\text{cells}\times \text{dilution}\;\text{factor}}{\text{Area}\times \text{thickness}\;\text{of}\;\text{liquid}\;\text{film}}.$$

### 2.12. Hematological Parameters

Blood was used for the estimation of hemoglobin (Hb) content, red blood cell (RBC) count, and white blood cell (WBC) count by standard procedures. 

### 2.13. Biochemical Estimation

Blood collected by cardiac puncture was used for the determination of SGPT, SGOT, and ALP. 

### 2.14. Statistical Analysis

All data are expressed as mean ± SEM (*n* = 6 mice per groups). Statistical significance (*p*) was calculated by one-way ANOVA between the treated groups and the EAC control followed by Dunnett's test of significance where *P* < .05 and *P* < .001 were considered to be significant and highly significant, respectively. 

## 3. Results

Intraperitonial administration of MECM at the dose levels of 200 and 400 mg/kg BW increased the life span, nonviable tumor cell count and decreased the tumor volume, viable tumor cell count as compared to the EAC control mice as shown in Table 1.

**Figure 1:**
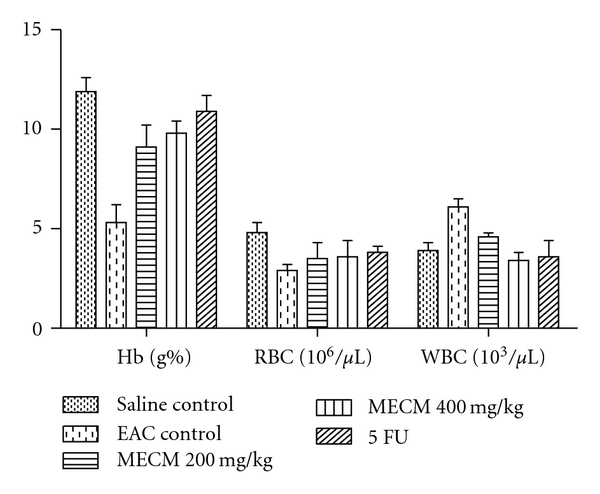
Effect of MECM on haematological parameters of EAC cell-bearing mice (*n* = 6). Experimental groups are compared with EAC control group (*P* < .001)

MECM also restored the haematological parameters towards normal level when compared with EAC control mice as shown in Figure 1.

**Figure 2:**
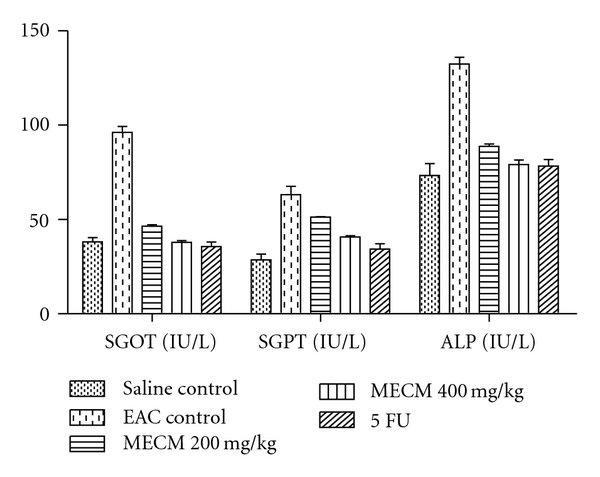
Effect of MECM on different biochemical parameters in EAC-treated mice (*n* = 6). Experimental groups are compared with EAC control group (*P* < .001).

The effects of MECM on different serum biochemical parameters are given in Figure 2. 

## 4. Discussion

The present study showed that MECM significantly decreased the WBC count and prolonged the life span of the EAC-treated groups as compared to those of the EAC control group. One of the major criteria of judging good anticancer drugs is that it should be able to prolong the life and decrease the leucocyte count [14]. Reduced volume of tumor and increased life span also indicated decrease of cell division [13]. The anaemia associated with carcinoma was also restored to normal levels when compared to the control group as indicated by the increased RBC count and hemoglobin level. These indicate that MECM has no toxic effects on the haematological system [15].

The serum biochemical parameters were restored to normal levels comparable to saline control indicating protection from the tumor cell-induced hepatotoxicity by MECM [16].

Preliminary phytochemical study indicated that MECM contained flavonoids, alkaloids, tannins, and saponin. In a previous study conducted by the authors the antioxidant activity of MECM was established against different reactive oxygen and nitrogen species [17].

The flavonoids and limonoids present in citrus plants are postulated to be the cause of their antitumor and anti-inflammatory effects [18]. Flavonoids have a chemopreventive role in cancer by means of their effect in signal transduction in cell proliferation and angiogenesis [13]. As mentioned earlier immature hexane fruit extract of pomelo was investigated for its antiproliferative activity and has already been proven to induce apoptosis against U937 human leukemia cell line [11], therefore a similar mode of action can be proposed for MECM.

Therefore it can be concluded from this study that *Citrus maxima *possesses potent anticancer activity which can be attributed to the flavonoids present in it which can therefore serve as a stepping stone for the discovery of a new anticancer agent. 

## Figures and Tables

**Table 1:** Effect of MECM on survival time, increased life span, tumor volume, viable cell count, and nonviable cell count of EAC tumor bearing mice (*n* = 6).

Parameters	EAC control	200 mg/kg MECL	400 mg/kg MECL	20 mg/kg 5-FU
Tumor volume (mL)	3.8 ± 0.26	2.15 ± 0.44*	1.56 ± 0.22*	0.73 ± 0.18*
MST (days)	19.5	28	34	40.5
%ILS	0	43.59	74.36	107.69
Viable cell ×10^7^	7.47 ± 0.14	1.71 ± 0.06*	1.39 ± 0.15*	0.93 ± 0.04*
Nonviable cell ×10^7^	0.32 ± 0.03	1.6 ± 0.04**	2.34 ± 0.05**	2.72 ± 0.05**
Total cell ×10^7^	7.79	3.31	3.73	3.65
Viable%	95.89	51.66	37.27	25.48
Nonviable%	4.11	48.34	62.73	74.52

*Experimental groups are compared with control group (*P* < .001).

**Experimental groups are compared with control group (*P* < .05).
